# Intraoperative Assessment of the Stromal Ablation in Photorefractive Keratectomy

**DOI:** 10.3390/jcm13071901

**Published:** 2024-03-25

**Authors:** Dana Barequet, Eliya Levinger, Nadav Levinger, Samuel Levinger, Irina S. Barequet

**Affiliations:** 1 Division of Ophthalmology, Tel Aviv Medical Center, Tel Aviv 6423906, Israel; eliya.levinger@gmail.com; 2 Tel Aviv Faculty of Medicine, Tel Aviv University, Tel Aviv 6934203, Israel; nadav.levinger@gmail.com (N.L.); dr.ronit@enaim.co.il (S.L.); ibarequet@yahoo.com (I.S.B.); 3 Enaim Refractive Surgery Center, Tel Aviv 6701101, Israel; 4 Goldschleger Eye Institute, Sheba Medical Center, Tel Hashomer, Ramat Gan 5262160, Israel

**Keywords:** photorefractive keratectomy, optical coherence pachymetry, stromal ablation, central ablation depth

## Abstract

**Purpose:** To evaluate the difference between planned and measured central ablation depth (CAD) and compare the first and second operated eye in simultaneous bilateral myopic alcohol-assisted PRK. **Methods:** A retrospective review of patients was performed. Demographic and preoperative data was abstracted. Intraoperative assessment included environmental data, laser-planned algorithm of ablation (L-CAD), and optical coherence pachymetry (OCP) measurements. The true stromal ablation depth (O-CAD) was calculated by subtracting the immediate post-ablation OCP measurement from the OCP measurement before laser ablation. Deviation in pachymetry (DP) between O-CAD and L-CAD was also assessed. **Results:** The study comprised 140 eyes from 70 consecutive patients. The mean age was 26.91 ± 6.52 years, and 57.1% were females. O-CAD was significantly correlated to preoperative refractive errors and intraoperative laser settings. DP was not correlated to any of the pre- or intraoperative parameters. L-CAD showed a significant underestimation as compared to O-CAD (67.87 ± 25.42 µm and 77.05 ± 30.79 µm, respectively, *p* < 0.001), which was shown in 74.3% of the cases. A moderate agreement between the two methods was noted, with a mean deviation of 17%. This difference was maintained for each eye individually (*p* < 0.001). In addition, DP was significantly higher in the first operated eye as compared to the second operated eye (11.97 ± 16.3 µm and 6.38 ± 19.3 µm respectively, *p* = 0.04). **Conclusion:** The intraoperative assessment of stromal ablation showed significantly higher central ablation depth values compared to the laser-planned ablation algorithm. The deviation in pachymetry was higher in the first, compared to the second, operated eye. Awareness is warranted as to the discrepancy between preoperative planning and intraoperative assessment.

## 1. Introduction

Corneal laser refractive surgery is a successful surgical treatment used for the reduction of refractive errors [1]. The advantages are submicron precision of the ablation, in which each pulse is pre-assumed to remove a constant amount of tissue. Corneal thickness plays a key role at all stages of a refractive correction [2]. The volume of tissue removal determines the refractive change, and corneal thickness provides structural support [3].

The ablation process cannot be adjusted after the initial programming starts. However, individual variations, stromal evaporation and swelling during laser treatment may diminish the accuracy of the results [3,4,5]. Ablations deeper than planned may lead to overcorrections and insufficient residual corneal thickness, which may increase the risk of postoperative keratectasia [6].

Methods for measuring corneal thickness, including ultrasound pachymetry and Scheimpflug imaging, are utilized in the diagnosis and follow-up of various corneal disorders as well as in the preoperative evaluation of refractive-surgery candidates [7]. The optical coherence pachymetry system (OCP, Heidelberg Engineering, Lübeck, Germany), integrated in the SCHWIND Amaris excimer laser, provides non-contact continuous measurements of the central corneal thickness during refractive procedures. It allows for intraoperative monitoring of the central corneal thickness throughout the entire surgery. The OCP has been shown to have high reproducibility of intraoperative corneal changes, such as the flap and the residual stromal thickness [8]. It has also been found to have a significant correlation between the measured and calculated ablation depth in photorefractive keratectomy (PRK) [9].

The assessment of stromal ablation depth is performed preoperatively in the surgical ablation planning by the laser algorithm, which predicts the central ablation depth (L-CAD); and intraoperatively by the OCP system, allowing for real-life central ablation depth (O-CAD). Previous studies reported an overestimation of O-CAD compared to other modalities such as ultrasound and Scheimpflug [10]. In addition, it has been shown that O-CAD estimation may be up to 29% higher than L-CAD [11,12]. These studies did not find a correlation between the deviation in the ablation process and the postoperative refraction. Other independent factors such as patient-specific data, preoperative shape of cornea, and environmental setup are believed to be influential in determining the volume of the ablation depth [13,14].

The purpose of our study is to evaluate the difference between planned and measured CAD and compare the first and second operated eye in simultaneous bilateral myopic alcohol-assisted PRK.

## 2. Materials and Methods

### 2.1. Patient and Study Design

This study was a retrospective evaluation of eyes that underwent bilateral simultaneous alcohol-assisted myopic PRK between October 2018 and September 2020. Indications for the PRK included eyes with corneal thickness greater than 480 µm, no contraindications for laser vision correction, and clinically indicated refractive errors being correctable with an aspheric PRK profile. We included patients with complete pre- and intraoperative data, as described below, and excluded from the analysis patients who underwent unilateral PRK and those missing complete data on both eyes. Patients received a detailed written informed consent form prior to surgery. The study complies with the Declaration of Helsinki and was approved by the Institutional Review Board at the Sheba Medical Center on 21 January 2019, approval code 5505-18-SMC.

### 2.2. Preoperative Examination

A detailed ophthalmic and systemic history was obtained. Contact lens wear was discontinued for at least 10 days, depending on contact lens type, prior to the final preoperative evaluation and surgery. Preoperative examination included uncorrected distance visual acuity (UDVA), best corrected distance visual acuity (BDVA), manifest and cycloplegic refraction, Scheimpflug tomography (Sirius, CSO, Florence, Italy) and central ultrasound pachymetry. A full ophthalmic examination was performed, including slit-lamp examination, intraocular pressure by Goldmann applanation tonometer measurement, corneal epithelium assessment by fluorescein staining, tear breakup time, Schirmer II testing, and dilated fundus examination.

### 2.3. Surgical Technique

All surgeries were performed, using the same technique, by two surgeons (I.S.B. and E.L.) using the SCHWIND Amaris 500E excimer laser platform (SCHWIND eye tech solutions GmbH, Kleinostheim, Germany). The OCP platform integrated in the laser system was utilized for the intraoperative central pachymetry measurements. The ablation algorithm was calculated using SCHWIND ORK-CAM software v.4.5 (Schwind Eye Tech-Solutions GmbH, Kleinostheim, Germany). The target refraction was emmetropia in all eyes.

In the preparation area, one drop of topical 0.4% oxybuprocaine hydrochloride (Localin, Fischer Pharmaceuticals Ltd., Bney Brak, Israel) and moxifloxacin (Vigamox, Alcon Laboratories, Inc., Fort Worth, TX, USA) were instilled in the eyes, and the lid margins were cleaned with a 5% povidone iodine solution. Immediately afterwards, the patient entered the laser room and moved to a supine position beneath the laser system. A sterile drape was placed at the lid margins and a lid speculum was inserted. The first OCP measurement of the corneal thickness was performed simultaneously with the alignment of the eye tracker on the eye. Another drop of topical 0.4% oxybuprocaine hydrochloride anesthesia was instilled in the operated eye, and then epithelial delamination was achieved with an 8.5 mm well placed centrally on the cornea and filled with 20% ethanol alcohol for 30 s, followed by absorption with a Merocel sponge and irrigation with sterile balanced salt solution. The epithelium was then debrided using a blunt spatula and the Bowman layer was exposed. A second OCP measurement was obtained prior to the laser ablation. The laser ablation was performed according to the preset programming. The third OCP measurement was recorded. Mitomycin C 0.02% was applied to the stromal bed for 10 to 30 s depending on the depth of the stromal ablation. The stromal bed was irrigated with 20 mL of chilled balanced salt solution, and a soft bandage contact lens was placed on the eye, to be kept for four to six days. A sterile transparent shield was placed on both eyes after completion of the surgical procedure. Postoperatively, the patients were instructed to use 0.5% moxifloxacin eye drops four times daily for seven days; 0.5% loteprednol eye drops (Lotemax, Bausch & Lomb, Inc., Rochester, NY, USA) four times daily for a month, and then tapered down gradually over the next month; and non-preserved artificial tears as needed. Patients were scheduled for postoperative examinations one day, one week, one month and three months postoperatively.

### 2.4. Data Collection

Data were abstracted for age, gender, contact lens wear, preoperative refractive errors, and keratometry (Javal keratometry). Central corneal pachymetry data were obtained as recorded from the ultrasound pachymetry and the tomographic parameter of central corneal thickness. Intraoperative data was recorded, including optical zone diameter (mm), transition zone (mm), ablation zone diameter (µm), ablation time (seconds), treatment time (seconds), temperature (°C), humidity (%), static and dynamic cyclotorsion correction, laser planned algorithm of central ablation depth (L-CAD) and intraoperative OCP measurements. The OCP measurements were recorded with first alignment of the eye (before epithelium removal), before laser ablation (after epithelium removal) and at the conclusion of laser ablation. The central epithelium thickness (ET) was calculated by subtracting OCP measurement after epithelium removal from the OCP measurement prior to the epithelium removal. The real-time stromal central ablation depth (O-CAD) was calculated by subtracting the immediate post-ablation OCP measurement from the OCP measurement before laser ablation. Deviation in pachymetry (DP) between O-CAD and L-CAD was also assessed.

### 2.5. Statistical Analysis

Data were recorded in Microsoft Excel 2010 and analyzed using SPSS version 25 (SPSS Inc., Chicago, IL, USA).

Continuous variables, such as stromal thickness, were compared between subjects using the independent sample *t*-test. In cases of paired variables, such as right and left comparison, data were restructured, and a paired sample *t*-test was used. Correlation of continuous variables was examined using Pearson’s correlation.

All tests were 2-tailed, and the threshold for statistical significance was defined as a *p*-value < 0.05.

## 3. Results

The study included 140 eyes of 70 consecutive patients. The mean age of the patients was 26.91 ± 6.52 years (range, 18 to 45 years), and 57.1% were females. Contact lens wear was reported for 58.6% of the eyes. The preoperative refractive measurements for all eyes included a mean sphere of −3.71 ± 1.82 D (range, −7.50 to −0.25 D); a mean cylinder of −0.68 ± 0.69 D (range, −5.00 to 0 D); and a mean spherical equivalent of -3.37 ± 1.80 D (range, −7.125 to +0.375 D). Table 1 demonstrates patients’ baseline characteristics.

Among the patients’ baseline characteristics, O-CAD was significantly correlated to preoperative refractive errors (Table 1). O-CAD was also significantly correlated to intraoperative laser and environmental settings (Table 2). There was no correlation to cyclotorsion parameters. The deviation of pachymetry (DP) between O-CAD and L-CAD was not correlated to any of the pre- or intraoperative parameters (Table 1 and Table 2).

**Figure 1:**
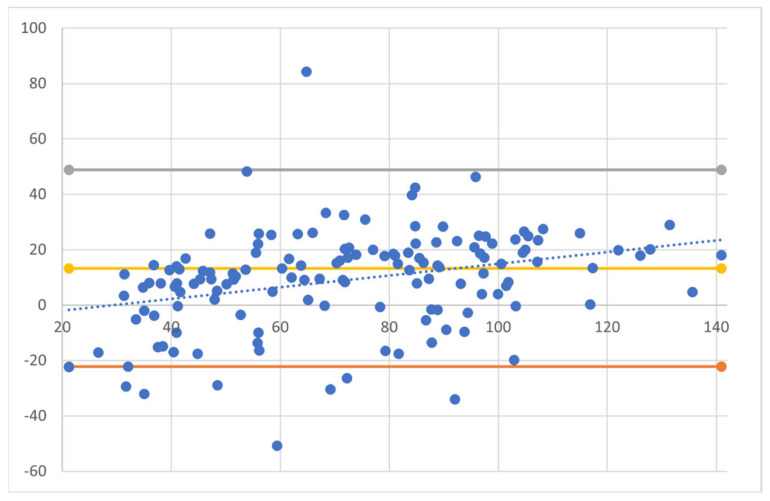
Bland–Altman plots showing the agreement between laser-planned central ablation depth (L-CAD) and OCP-obtained central ablation depth (O-CAD). A moderate agreement was noted between the L-CAD and O-CAD, with the limits of agreements ranging from −22.3 µm to +48.8 µm. Dotted trendline expressing minimal proportion bias.

Comparison between L-CAD and O-CAD showed a significant underestimation of the laser-planned CAD, as compared to real-life OCP-measured CAD (67.87 ± 25.42 μm and 77.05 ± 30.79 μm, respectively, *p* < 0.001). Bland–Altman plots show the agreement among the two measurement methods (Figure 1). A moderate agreement was noted between the L-CAD and O-CAD, with a mean deviation from nominal values of 16.64 μm (17%), with the limits of agreements ranging from −22.3 µm to +48.8 µm. The plot demonstrates an underestimation of L-CAD as compared to O-CAD in 74.3% of the cases.

**Figure 2:**
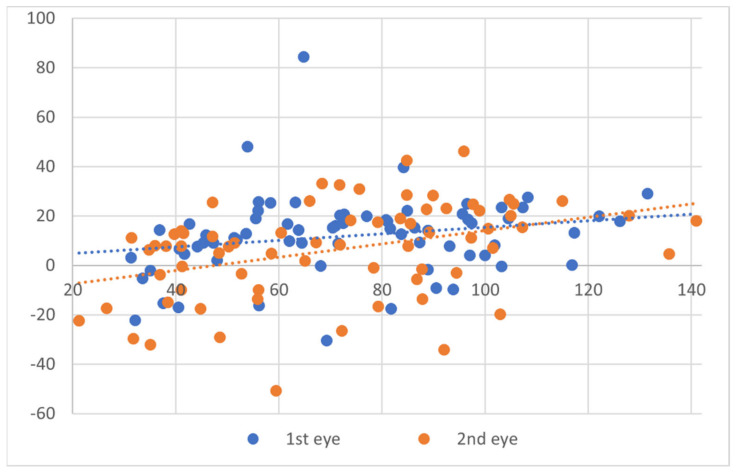
Bland–Altman plots showing the agreement between laser-planned central ablation depth and OCP-obtained central ablation depth for each operated eye. Dotted trendline expressing minimal proportion bias.

**Figure 3:**
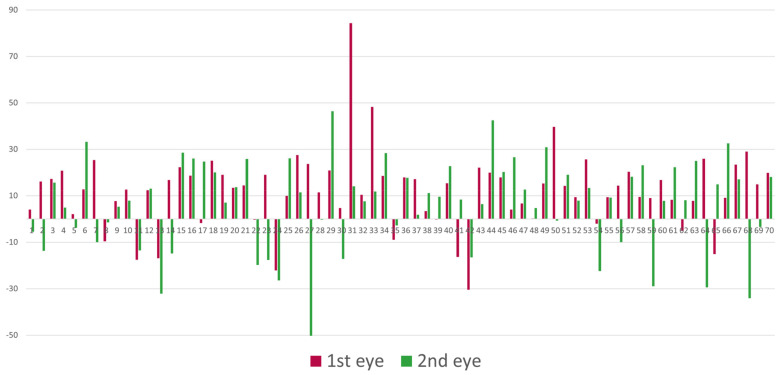
Deviation in pachymetry for the first- and second-operated eye of each patient.

When comparing the Bland–Altman plots for each operated eye, significant differences between the two measurement methods were observed for each eye individually (first operated eye, *p* < 0.0001; second operated eye, *p* < 0.001) (Figure 2). In addition, DP was significantly higher for the first operated eye as compared to the second operated eye (11.97 ± 16.3 µm and 6.38 ± 19.3 µm respectively, *p* = 0.04). Figure 3 demonstrates the DP in the first- and second-operated eye of each patient, showing a higher DP in the first eye for the majority of patients (56%).

Postoperative refractive errors and outcomes at 3 months are shown in Table 3. There is no difference in UDVA and BDVA between the two fellow operated eyes. A small yet significant difference in sphere can be observed between the fellow eyes (first operated eye: +0.25 ± 0.3 D; second operated eye: +0.32 ± 0.4 D, *p* = 0.03).

## 4. Discussion

In this study, we investigated the deviation of ablation pachymetry between intraoperative OCP measurements and laser planned stromal ablation depth in eyes undergoing myopic alcohol-assisted PRK.

Optical coherence pachymetry integrated in the SCHWIND Amaris excimer laser provides intraoperative central corneal thickness measurements during refractive procedures. By recording specific measurements at exact time points during the surgery (i.e., prior to the surgery, prior and after epithelium removal, and after the stromal ablation) one can assess the real-time thicknesses of the various layers. This may improve safety by providing insight into the physical changes of the cornea during surgery, monitoring corneal thickness and ensuring an adequate residual stromal bed. In our study, the OCP showed significantly higher central ablation depth values compared to the planned L-CAD (*p* < 0.001), with a mean deviation of 17%. This is in agreement with previous studies, which have found the deviation in ablation to be between 11–29% in depth [8,10,11,12]. Adib-Moghaddam S et al., [12] studied the accuracy of central ablation depth compared to online pachymetry results in eyes undergoing transPRK. They found a significant difference between pre-assumed CAD and the amount of ablation measured by OCP, with a deviation of 28% from nominal values. Wirbelauer C et al. also evaluated intraoperative ablation parameters during myopic and hyperopic laser-assisted in situ keratomileusis, and found the intraoperative ablation to be up to 29% higher than in the planning setting.

The difference in the planned-versus-measured ablation depths may be partially explained by intraoperative corneal changes. This includes both the swelling effect of topical anesthesia and the corneal dehydration during laser ablation [4,8,15]. Clinical and experimental studies confirmed a significant corneal dehydration resulting in stromal thinning of up to 0.3 μm per second [4,11,15]. The toxic effect of topical anesthesia has also been established, ranging from a decrease of more than 10 µm to an increase of over 30 µm in individual cases [16,17]. OCP provides continuous measurement during the laser ablation and likely incorporates these effects of dehydration, which would increase the volume of tissue ablated. However, these changes are not predicted in the preoperative planning. Interestingly, neither intraoperative ambient temperature and humidity levels nor treatment and ablation times were correlated to DP. Thus, the intraoperative role of corneal hydration remains not very well understood.

We also found that the deviation in pachymetry was greater for the first operated eye compared to that of the second operated eye (*p* = 0.04). This difference, to the best of our knowledge, has not been previously described, and may be attributed to the time elapsed from the beginning of surgery as the toxic swelling effect of the anesthetic would probably be greater in the second operated eye. Penna et al. [18] demonstrated with oxybuprocaine that when the anesthetic diffuses deep into the stroma, it may inhibit endothelial cell metabolism, which leads to corneal edema. Weekers et al., [19] in a study on the influences of cocaine, lidocaine, and benoxinate, concluded that topical anesthetics caused an alteration of the Na^+^/K^+^ endothelium pump, resulting in increased osmotic pressure in the cornea and subsequent increased hydration of the stroma.

Not surprisingly, O-CAD was correlated to preoperative refractive errors and laser setting parameters, as the depth of ablation is derivative of the refractive errors, as well as affecting parameters such as ablation time and zone.

As previously shown [10,11,12], the relation between the pre-assumed ablation depth and the intraoperative OCP measurement technique was limited by predicting the achieved refractive outcome. This is because the major determinant of corneal refractive power is the corneal curvature, not the change in corneal thickness.

The limitations of our study are related to its retrospective nature, although by collecting data on consecutive eyes, we attempted to minimize collection bias. Another limitation is related to the indirect assessment of ET and central stromal ablation depth values, calculated by subtracting OCP measurements before and after epithelium removal and before and after completing the excimer laser stromal ablation, respectively. However, the measurements were performed at the same settings and eye tracking alignments, thus minimizing setting errors.

In summary, this study confirms that the intraoperative corneal changes in PRK may alter the preoperative planned stromal ablation depth. We showed that the intraoperative assessment of stromal ablation provided a significantly higher central ablation depth values compared to the laser-planned ablation algorithm. This deviation in pachymetry was significantly higher in the first compared to the second operated eye. OCP provides continuous real-time monitoring of corneal thickness and ensures an adequate residual stromal bed. However, the limitation of the current OCP system is the acquisition of a single point in the center without correcting for real-time individual changes. Further development may expand the role of OCP from continuous monitoring to the active control of individual excimer laser ablation and thus contribute to improved safety standards during refractive surgery.

## Figures and Tables

**Table 1:** Association between O-CAD and DP as to demographics and preoperative measurements. O-CAD, optical coherence pachymetry measured central ablation depth; DP, deviation in pachymetry. Bold represents significant results.

	Patients *n* = 70	Eyes *n* = 140	O-CAD		DP	
			*p*	r_p_	*p*	r_p_
Age, years (mean ± SD)	26.91 ± 6.52		0.78	0.02	0.18	−0.11
Gender-female, *n*(%)	40 (57.1)		0.97		0.95	
History of contact lens, %		58.6	0.80		0.73	
Refractive errors, D (mean ± SD)						
Sphere		−3.71 ± 1.82	**<0.001**	−0.73	0.53	0.06
Cylinder		−0.68 ± 0.69	**<0.001**	−0.36	0.88	0.02
Spherical equivalent		−3.37 ± 1.80	**<0.001**	0.67	0.54	0.06
Keratometry, D (mean ± SD)						
K1		43.44 ± 1.56	0.63	0.05	0.23	0.11
K2		44.36 ± 1.57	0.06	0.17	0.11	0.15
K average		43.90 ± 1.53	0.23	0.11	0.15	0.13
Preoperative pachymetry, µm (mean ± SD)						
US pachymetry		540.17 ± 29.57	0.82	0.02	0.14	−0.14
Topography (Sirius)		543.12 ± 30.99	0.87	−0.02	0.26	−0.11
Optical coherence pachymetry		536.30 ± 35.16	0.78	0.02	0.86	−0.02

**Table 2:** Association between O-CAD and DP as to intraoperative measurements and laser settings. Data is shown in mean ± SD. L-CAD, laser-planned central ablation depth; O-CAD, optical coherence pachymetry measured central ablation depth; DP, deviation in pachymetry; SCC, static cylcotorsion control; DCC, dynamic cyclotorsion control. Bold represents significant results.

	Eyes *n* = 140	O-CAD		DP	
		*p*	r_p_	*p*	r_p_
L-CAD, µm	67.87 ± 25.42	**<0.0001**		0.73	−0.03
O-CAD, µm	77.05 ± 30.79			**<0.0001**	0.57
DP, µm	9.18 ± 18.12	**<0.0001**	0.57		
Optical zone, mm	6.42 ± 0.28	**<0.0001**	−0.45	0.90	−0.01
Transition zone, mm	1.32 ± 0.35	**<0.0001**	0.80	0.80	−0.02
Ablation zone, µm	7.79 ± 0.68	0.14	0.13	0.49	−0.06
Ablation time, s	14.61 ± 5.98	**<0.0001**	0.80	0.91	−0.01
Treatment time, s	15.03 ± 6.57	**<0.0001**	0.79	0.83	0.02
Temperature, °C	25.50 ± 0.80	**<0.0001**	−0.36	0.15	−0.12
Humidity, %	35.68 ± 4.91	**<0.0001**	0.40	0.92	0.01
SCC, °	0.91 ± 4.03	0.59	−0.05	0.81	−0.02
DCC min, °	−0.58 ± 0.76	0.52	−0.06	0.89	−0.01
DCC max, °	0.84 ± 0.94	0.64	0.04	0.18	−0.11
Laser setting refractive errors, D					
Sphere	−3.83 ± 1.79	**<0.0001**	−0.73	0.90	0.01
Cylinder	−0.67 ± 0.60	**<0.0001**	−0.32	0.56	0.05
Spherical equivalent	−3.50 ± 1.79	**<0.0001**	−0.68	0.98	0.00

**Table 3:** Postoperative refractive errors and outcomes. Data is shown in mean ± SD. UDVA, uncorrected distance visual acuity; BDVA, best corrected distance visual acuity.

	All Eyes *n* = 140	1st Eye *n* = 70	2nd Eye *n* = 70	*p*
UDVA, decimal	+0.94 ± 0.2	+0.94 ± 0.2	+0.95 ± 0.2	0.8
BDVA, decimal	+1.01 ± 0.1	+1.01 ± 0.1	+1.02 ± 0.1	0.7
Sphere, D	+0.29 ± 0.3	+0.25 ± 0.3	+0.32 ± 0.4	0.03
Spherical equivalent, D	+0.40 ± 0.5	+0.36 ± 0.4	+0.45 ± 0.5	0.07

## Data Availability

Data are contained within the article.
